# Comparison of Growth and Water Quality in the Cultivation of *Anguilla japonica* and *Lactuca sativa* in Aquaponics with Biofloc and RAS Technologies

**DOI:** 10.3390/ani15243591

**Published:** 2025-12-14

**Authors:** Ju-ae Hwang, Jun Seong Park, Hae Seung Jeong

**Affiliations:** Advanced Aquaculture Research Center, National Institute of Fisheries Science, Changwon 51688, Republic of Korea; hjuae1031@korea.kr (J.-a.H.); pjs5420939@gmail.com (J.S.P.)

**Keywords:** *Anguilla japonica*, aquaponics, biofloc technology (BFT), recirculating aquaculture system (RAS), sustainable aquaculture, water characteristic

## Abstract

Sustainable aquaculture requires innovative systems that enhance production efficiency while minimizing environmental impact. Among emerging technologies, biofloc technology (BFT) and recirculating aquaculture Systems (RAS) are widely adopted for intensive fish culture; however, their integration with aquaponics remains underexplored. This study aimed to compare the growth performance and water quality in aquaponic (AP) systems combining Japanese eel (*A. japonica)* and caipira lettuce (*L. sativa*) using BFT and RAS technologies for 28 days. This study compared four aquaculture configurations: BFT, RAS, BFT-AP (BFT with aquaponics), RAS-AP (RAS with aquaponics). Results showed that aquaponics integration seems to be improve the fish growth and apparent feed efficiency. Eels reared in BFT-AP achieved the highest final weight, weight gain, specific growth rate and apparent feed efficiency, which were relatively greater than in BFT, RAS, or RAS-AP systems. The growth of caipira lettuce was markedly enhanced in BFT-AP, with total biomass nearly four times that of RAS-AP. Electrical conductivity and total dissolved solids were relatively higher in BFT-AP than in RAS-AP, indicating nutrient enrichment beneficial for plants. In conclusion, integrating aquaponics with BFT substantially enhanced both eel and production of caipira lettuce. The BFT-AP configuration represents a more efficient and ecologically balanced model for sustainable aquaculture intensification compared with traditional RASs.

## Simple Summary

This study investigated the effects of integrating aquaponics into biofloc (BFT) and recirculating aquaculture systems (RAS) on the growth performance of Japanese eel (*Anguilla japonica*), caipira lettuce (*Lactuca sativa*), and water quality. After 4 weeks of rearing, eels in aquaponics-integrated groups (BFT-AP and RAS-AP) showed relatively higher growth rates compared to those in conventional systems (BFT and RAS). The biomass of caipira lettuce was greater in BFT-AP than in RAS-AP. Higher electrical conductivity (EC) and total dissolved solids (TDS) in BFT-AP water indicated nutrient enrichment beneficial for plant growth, without negatively affecting fish performance. These findings suggest that integrating aquaponics, particularly with BFT, can enhance both eel and plant productivity while maintaining water quality sustainability.

## 1. Introduction

The Food and Agriculture Organization of the United Nations (FAO) introduced the Blue Transformation framework (2022–2030) as a strategic vision to enhance the role of aquatic food systems in global food security and nutrition [1]. This initiative promotes the coordinated use of existing and emerging technologies by governments, agencies, and communities to sustainably maximize the contribution of marine and inland aquatic resources to healthy and affordable diets [1]. According to the Blue Transformation roadmap, the main objective for aquaculture is “sustainable aquaculture intensification and expansion that satisfies global demand for aquatic food and distributes benefits equitably.” Among the five targets defined for aquaculture development, one emphasizes the need for operations that minimize environmental impacts and use resources efficiently [1].

Globally, aquaculture production has grown from 21.8 million tonnes in the 1990s to 87.5 million tonnes in 2020, while capture fisheries have remained nearly static, increasing only from 88.9 to 90.3 million tonnes during the same period [2]. Per capita consumption of aquatic foods rose to 20.2 kg in 2020, more than double the 9.9 kg recorded in the 1960s [2]. As wild capture production reaches its natural limits, aquaculture has become the primary means to meet the growing global demand for aquatic products. However, without sustainable management, aquaculture expansion can lead to water pollution, resource depletion, and ecological imbalance [3]. Thus, the rapid intensification of aquaculture has heightened global concern over its environmental sustainability. To address these challenges, innovative and eco-friendly systems such as recirculating aquaculture systems (RAS), biofloc technology (BFT), and aquaponics are being developed and adopted.

The RAS is a closed-loop technology designed to culture aquatic organisms by continuously reusing treated water. Mechanical and biological filtration units remove or convert waste products such as solids, ammonia, and carbon dioxide into non-toxic forms before the purified, oxygen-enriched water is returned to the culture tanks [4]. This system allows precise environmental control, enabling predictable growth rates, efficient feed utilization, and stable production schedules.

BFT has attracted increasing attention over the past two decades as an environmentally responsible and economically viable aquaculture approach [5]. It relies on the establishment of a microbial community within the culture system that provides ecosystem services such as nutrient recycling and supplemental nutrition for cultured species. Proper management of inputs—including feed, carbon sources, aeration, and microbial balance—enables efficient nutrient utilization, stable water quality, and improved cost-effectiveness.

Aquaponics integrates aquaculture with hydroponic plant cultivation in a recirculating system [6]. In these systems, metabolic by-products from fish culture are transformed into nutrients that support plant growth, reducing waste discharge and enhancing resource use efficiency. When aquatic and terrestrial crops are co-cultured in this closed-loop design, the system is termed aquaponics [7]. Such integration promotes circular resource flow and environmental sustainability while generating additional economic value.

The Japanese eel (*A. japonica*) is a high-value species widely consumed in East Asia, particularly in China, Japan, and Republic of Korea, owing to its superior nutritional and economic importance. In Korea, Japanese eel (*A. japonica*) represent the most important inland aquaculture species, with an annual production of approximately 16,058 tonnes and an estimated market value of 514 billion KRW, accounting for about 82% of the national inland aquaculture output value in 2024 [8].

Given these developments, considerable research has investigated the application of RAS, BFT, and aquaponic systems in various freshwater fish species, including catfish and *Anguilla* spp. [9,10,11,12,13,14,15,16,17,18,19,20]. It was reported that using biofilm–biofloc in in situ aquaculture water treatment, the water saving and pollution reducing advantages were remarkable, the aquaculture water quality was improved, and the growth of *Anguilla marmorata* was greatly promoted [9]. Recently, the term “FLOCponics” has been use to describe integrated systems combining BFT with aquaponics, and several studies have reported enhanced nutrient availability and plant growth in such systems [18,19,20]. Park et al. [20] observed enhanced growth of *Silurus asotus* and *Anguilla bicolor* in FLOCponics compared to conventional BFT or flow-through systems. However, no comparative studies have yet examined aquaponic integration in BFT and RASs. Therefore, present study aims to evaluate the growth performance of Japanese eels (*A. japonica*) and caipira lettuce (*L. sativa*) aquaponics applied to both BFT and RAS, focusing on water quality parameters and major ion and nutrient concentration.

## 2. Materials and Methods

### 2.1. Experimental System Setup and Conditions

**Figure 1:**
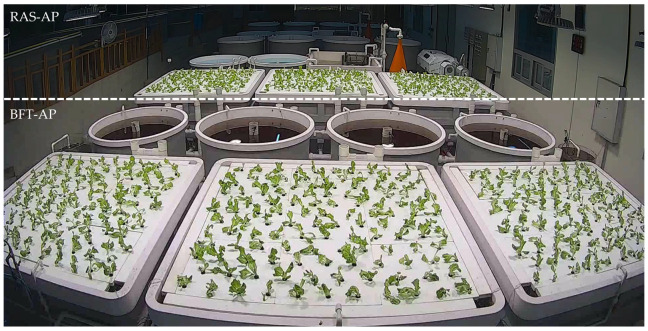
Overall layout of BFT-AP and RAS-AP systems after planting.

Four experimental groups were established: BFT, RAS, BFT-AP, and RAS-AP. The entire experiment lasted 72 days, consisting of 30 days for microbial inoculation, 14 days for plant seedling preparation, and 28 days for fish and plant rearing. A 2 × 2 factorial design was employed, with two fixed factors: (1) culture system (BFT or RAS) and (2) aquaponic application (with or without AP). A total of sixteen circular fiber-reinforced plastic (FRP) tanks (diameter: 1.5 m; height: 0.6 m; volume: 1.06 m^3^) were used to culture Japanese eels (*A. japonica*), with four replicate tanks assigned to each treatment. In aquaponic treatments (BFT-AP and RAS-AP), each fish tank was connected to three rectangular FRP plant beds (width: 2.0 m; length: 2.0 m; height: 0.6 m; volume: 2.4 m^3^), resulting in six plant beds (three per treatment). Fish were randomly allocated among tanks to minimize potential bias (Figure 1).

**Figure 2:**
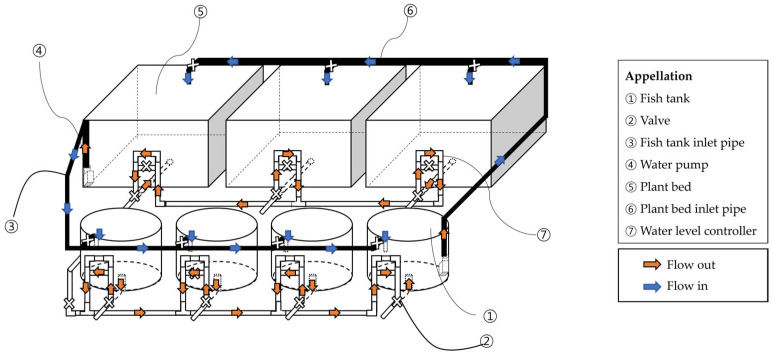
Schematic diagram of BFT-AP system.

**Figure 3:**
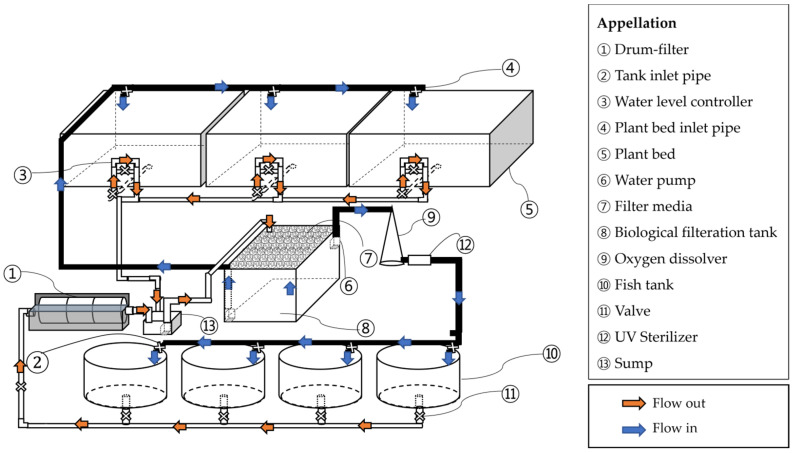
Schematic diagram of RAS-AP system.

In BFT group, four rearing tanks were operated independently, each with a separate aeration and water circulation system. In RAS and RAS-AP treatments, four rearing tanks shared a common recirculating loop consisting of a drum filter (60 µm), a moving-bed biofilter (40% fill ratio), a UV sterilizer, and an oxygen dissolver. The water turnover rate of RAS was maintained at approximately 1 h^−1^. In BFT-AP and RAS-AP systems, three plant beds (1.0 m^2^ each) were connected to the shared loop. Valves were installed to isolate each tank for draining and sampling, but tanks within the same loop were not considered independent replicates. Therefore, data were analyzed descriptively (mean ± SD) (Figure 2 and Figure 3).

During rearing, the carbon-to-nitrogen (C/N) ratio in BFT and BFT-AP systems was maintained at 15:1. Both systems were strongly aerated to prevent biofloc sedimentation and solids using an air blower (Hi-blow HP-80, Techno Takatsuki Co., Ltd., Osaka, Japan) and a 25 W water pump (Hyupsin, Seoul, Republic of Korea). Additional oxygen was supplied using an oxygen generator (KMOS-40Rl, Kumho Marine, Busan, Republic of Korea). Each plant bed received gentle aeration to prevent floc accumulation around the roots. The water flow rate from fish tanks to plant beds was adjusted to two complete cycles per day. No water exchange was performed during the entire experimental period in any system (BFT, BFT-AP, RAS, or RAS-AP).

A total of 720 Japanese eels (initial weight: 110.5 ± 0.5 g, a density of 5.0 ± 0.0 kg m^−3^) were randomly distributed into 16 FRP tanks (45 individuals per tank), providing four replicates for each treatment. Fish were fed a commercial extruded pellet (EP; National Federation of Fisheries Cooperatives Feed, Uiryeong-gun, Gyeongsangnam-do, Korea) containing 52% crude protein, 10% crude lipid, 3% crude fiber, and 15% crude ash. Feed was supplied to apparent satiation twice daily (09:30 and 16:30), corresponding to approximately 2–3% of total biomass per day. Molasses (as a carbon source for BFT and BFT-AP) was added 1 h after final feeding (17:30). The water temperature was maintained at 26.0 ± 0.5 °C using a 1 kW heater (OKE-HE-100, Sewon OKE, Suwon, Republic of Korea).

### 2.2. Water Preparation and Rearing Management

The BFT water was prepared according to the method of Park et al. [20]. Biofloc volume was quantified using Imhoff cones. One liter of water was collected from each tank, allowed to settle for 15 min, and the settled volume (mL/L) was recorded as an indicator of biofloc accumulation and the mean volume of biofloc was 15.5 mL/L during experimental periods

The target C:N ratio of 15:1 was maintained based on nitrogen input from feed. The required carbon input was calculated using the formula: Carbon required (g) = [Feed input (g) × 0.16 × C:N target]/carbon fraction of molasses, following Avnimelech [20]. Molasses (≈40% carbon) was added daily after feeding to sustain heterotrophic microbial activity. Sludge was removed periodically, and microscopic observations confirmed mature floc structures.

The rearing trial commenced once the concentrations of total ammonia nitrogen (TAN) and nitrite nitrogen (NO_2_^−^-N) peaked and subsequently declined to approximately 1 mg/L. Water temperature was maintained at 26.0 ± 0.5 °C throughout the trial. After acclimation, the fish were distributed into experimental tanks, and water was allocated evenly to each tank and plant bed.

### 2.3. Aquaponics

Two types of aquaponic systems were established: BFT-AP and RAS-AP, each with three replicated plant beds. All plant beds used deep water culture (DWC) method and contained 120 pots per bed (30 plants m^2^) in a coupled configuration. Indoor cultivation was supported by LED lighting under a 12L:12D photoperiod with PPFD of 200 µmol m^−2^s^−1^, maintaining an average light intensity of >6000 lux for optimal photosynthesis. No external nutrient supplementation was added during the trial. The water flow rate and turnover rate between fish tanks and plants beds were maintained at approximately 100 L/h and 0.04 h^−1^, respectively.

Caipira lettuce seeds (Enza Zaden, Enkhuizen, The Netherland) were sown in terra-plug (Smithers-Oasis Co., Ltd., Kent, OH, USA) and germinated in a controlled chamber (24 °C) at the Advanced Aquaculture Research Center. After 14 days, when seedlings reached the true-leaf stage (four days post-germination), 120 seedlings (2.0 ± 0.47 g) were transplanted into each plant bed.

### 2.4. Water Quality Analysis

Water quality was monitored daily using a YSI 650 Multiparameter Display System (YSI Incorporated, Yellow Springs, OH, USA) to measure temperature (°C), dissolved oxygen (DO, mg/L), pH, electrical conductivity (EC, mS/cm), and total dissolved solids (TDS, mg/L). Measurements were taken at 09:30 before the morning feeding. When pH dropped below 6.5, sodium bicarbonate was added for adjustment.

Nitrogen compounds including TAN, NO_2_^−^-N, and NO_3_^−^-N were analyzed using commercial test kits (Merck KGaA, Darmstadt, Germany) and an absorptiometer following the three-dust-spot method, a standardized colorimetric procedure described in the Merck technical manual for nitrogen determination. At the end of experiment, water samples were stored at −80 °C for further chemical analyses. Total nitrogen was determined using a Kjeltec 2300 analyzer (FOSS Tecator, Hillerød, Denmark), total phosphate by UV spectrophotometry (UV2450, Shimadzu, Kyoto, Japan), and elemental concentrations (K, Ca, Mg, Fe, Cu, Zn, Si) by inductively coupled plasma optical emission spectrometry (Optima 8300, PerkinElmer, Waltham, MA, USA). Chloride (Cl^−^) and sulfate (SO_4_^2−^) were quantified using ion chromatography (930 Compact IC Flex, Metrohm Co., Herisau, Switzerland).

### 2.5. Growth Performance and Production of Fish and Corps

At the end of trial, thirty fish were randomly sampled from each tank and anesthetized with 100 ppm MS-222 (Sigma-Aldrich, St. Louis, MO, USA) to measure body weight and total length. Growth performance indices were calculated as follows:

Survival (%) = (number of fish at the end of trial/number of fish at the beginning of trial) × 100,(1)

Weight gain (WG; g/fish) = final body weight–initial body weight,(2)

Specific growth rate (SGR; %/day) = [(ln final weight of fish − ln initial weight of fish)/days of trial] × 100,(3)

Condition factor (CF; g/cm^3^) = body weight (g) × 100/total length (cm)^3^, (4)

Apparent Feed efficiency (FE) = weight gain of fish/feed consumed.(5)

After 4 weeks, ten plants from each bed were randomly collected to record total weight, shoot length, leaf weight, and leaf length. Plant weight was measured using an electronic balance (MW-200, CAS, Seoul, Republic of Korea; ±0.01 g), and leaf dimensions were measured using digital Vernier calipers (Mitutoyo, Kawasaki, Japan; ±0.1 mm).

### 2.6. Statistical Analysis

The RAS, RAS-AP, and BFT-AP systems each operated under a single hydraulic loop, and tanks within the same loop shared common filtration and plant-bed water, limiting their complete statistical independence. Therefore, tanks were considered subsamples (technical replicates) rather than independent experimental units. Accordingly, inferential statistics such as two-way ANOVA were not applied at the tank level; instead, descriptive statistics (mean ± SD) were used to illustrate biological trends and system-level responses across treatments. For statistical purposes, the system loop itself was considered the experimental unit (n = 1 per system), and all tank and plant bed measurements were regarded as subsamples. The results were interpreted cautiously, focusing on system-level behavior rather than population-level inference.

## 3. Results

### 3.1. Growth Performance of Fish and Plants

Table 1 presents the growth performance of eels after the 4-week trial. The mean survival rate were 91.1–100.0% and lower in RAS-AP groups compared to the other groups. The mean final weight, weight gain and specific growth rate (SGR) were higher in the aquaponics groups (BFT-AP and RAS-AP) compared to the non-aquaponics groups (BFT and RAS), particularly in BFT-AP treatment. The mean condition factor (CF) of eels was similar across treatment. The mean apparent feed efficiency (FE) were higher in BFT and BFT-AP group, particularly in BFT-AP treatment.

**Figure 4:**
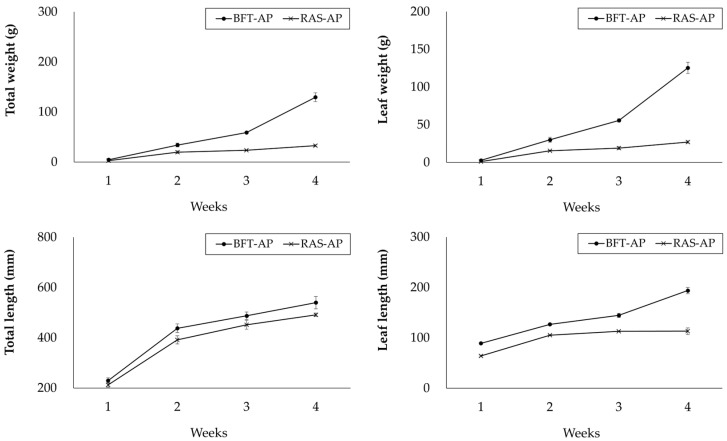
Growth performance of caipira lettuce (*L. sativa*) in BFT-AP and RAS-AP for 4 weeks. Values are presented mean ± SD of three subsample plant bed within each shared system (system = 1). No inferential statistical tests were performed due to the shared system structure.

Figure 4 presents the growth performance of caipira lettuce in BFT-AP and RAS-AP during the 4-week trial. The mean total weight and leaf weight of caipira lettuce were higher in BFT-AP group. Total weight of caipira lettuce rearing in BFT-AP and RAS-AP were 129.4 ± 8.8 g and 32.6 ± 1.7 g, respectively. Leaf weight of caipira lettuce rearing in BFT-AP and RAS-AP were 125.5 ± 7.4 g and 27.1 ± 1.5 g, respectively.

Total length and leaf length of caipira lettuce were similar across treatment. Total length of caipira lettuce rearing in BFT-AP and RAS-AP were 539.8 ± 24.5 mm and 491.4 ± 6.6 mm, respectively. Leaf length of caipira lettuce rearing in BFT-AP and RAS-AP were 193.9 ± 6.2 mm and 113.2 ± 6.4 mm, respectively.

### 3.2. Water Qualtiy

**Figure 5:**
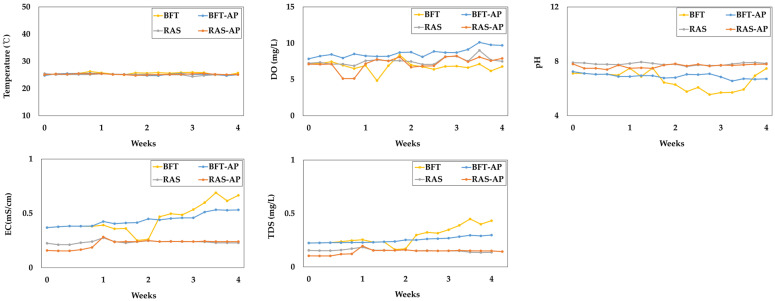
Basic water quality parameters during the 4-week experiment.

The variation in basic water quality parameters such as, temperature, dissolved oxygen (DO), pH, electrical conductivitiy (EC), total dissolved solids (TDS) were presented in Figure 5. The basic water parameters for BFT, BFT-AP, RAS, and RAS-AP experimental groups were as follows:

Temperature (°C) = 25.54 ± 0.40, 25.18 ± 0.30, 25.10 ± 0.23, and 25.22 ± 0.19;
DO (mg/L) = 6.61 ± 0.68, 8.68 ± 0.64, 7.55 ± 0.51, and 7.22 ± 0.90 mg/L;
pH = 6.61 ± 0.68, 6.91 ± 0.18, 7.80 ± 0.09, and 7.65 ± 0.14;
EC (mS/cm) = 0.45 ± 0.13, 0.44 ± 0.06, 0.24 ± 0.01, and 0.22 ± 0.04;
TDS (mg/L) = 0.29 ± 0.09, 0.25 ± 0.03, 0.15 ± 0.01, and 0.14 ± 0.02, respectively.

**Figure 6:**
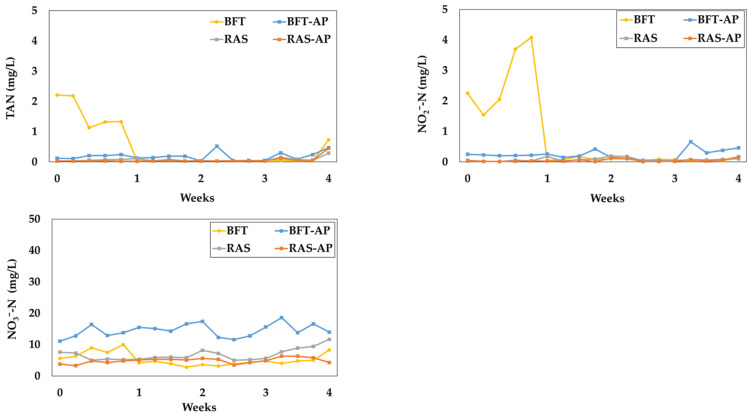
Nitrogen-related water quality parameters during the 4-week experiment.

The variation of nitrogen-related water quality parameters such as, total ammonia nitrogen (TAN), NO_2_^−^-N and NO_3_^−^-N were presented in Figure 6. The nitrogen-related water parameters for BFT, BFT-AP, RAS, and RAS-AP experimental groups were as follows:

TAN (mg/L) = 0.52 ± 0.77, 0.18 ± 0.14, 0.07 ± 0.06, and 0.06 ± 0.10;
NO_2_^−^-N (mg/L) = 0.82 ± 1.33, 0.24 ± 0.16, 0.07 ± 0.06, 0.05 ± 0.05;
NO_3_^−^-N (mg/L) = 5.33 ± 2.07, 14.51 ± 2.10, 6.21 ± 2.44, 4.89 ± 0.86, respectively.

### 3.3. Major Ion and Nutrient Concentration of Rearing Water and Plant

Table 2 presents the major ion and nutrient concentrations of rearing water after 4-week experiment in BFT, BFT-AP, RAS, RAS-AP systems. All major ion and nutrient concentrations of rearing water in BFT and BFT-AP were relatively higher than RAS and RAS-AP group, except for Si.

Table 3 presents the major ion and nutrient concentrations of caipira lettuce (*L. sativa*) after 4-week experiment in BFT-AP and RAS-AP systems. All major ion and nutrient concentrations of caipira lettuce in BFT-AP were higher than RAS-AP group, except for Si. BFT-AP and RAS-AP experimental groups were as follows (mg/L): Cl = 25.85 ± 0.75 and 17.90 ± 1.56; SO_4_ = 22.50 ± 0.57 and 18.05 ± 7.14; Total-N = 5.31 ± 1.99 and 4.56 ± 0.60; Total-P = 3.46 ± 0.16 and 0.30 ± 0.12; Ca = 33.35 ± 1.48 and 26.15 ± 0.07; Cu = 0.03 ± 0.00 and 0.01 ± 0.00; Fe = 0.07 ± 0.01 and 0.02 ± 0.00; K = 2.85 ± 0.07 and 0.50 ± 0.00; Na = 40.05 ± 1.48 and 17.35 ± 0.07; Si = 2.03 ± 0.09 and 2.12 ± 0.05.

## 4. Discussion

The Blue Transformation Roadmap proposed by the FAO identifies the primary objective of aquaculture as achieving “sustainable aquaculture intensification and expansion that satisfies global demand for aquatic food and distributes benefits equitably.” To realize this goal, aquaculture must operate with minimal environmental impact and efficient resource use [1]. Therefore, sustainable systems such as recirculating aquaculture systems (RAS), biofloc technology (BFT), and aquaponics are increasingly being emphasized as key solutions. In present study, Japanese eels (*A. japonica*) were cultured under BFT and RAS conditions, with and without aquaponic integration, to evaluate growth performance, plant productivity, and water quality.

Eels in aquaponics-applied systems (BFT-AP and RAS-AP) were higher in final weight, WG, and SGR than those in non-aquaponic systems, suggesting that the integration of aquaponics can improve fish growth. Similar findings were reported by Park et al. [20] who observed enhanced growth of Japanese eel (*A. japonica*), Far Eastern catfish (*S. asotus*), and tropical eel (*Anguilla bicolor*) in FLOCponics compared to conventional BFT or flow-through systems. Hwang et al. [18] also reported that the improved growth in FLOCponics may be attributed to the presence of plants acting as natural biofilters, enhancing microbial balance and promoting beneficial bacteria such as *Bacillus* spp. and PAC000036_g [18], which contribute to better water quality and nutrient recycling.

The apparent feed efficiency (FE) was higher in eels reared in BFT and BFT-AP systems compared with RAS and RAS-AP. This aligns with studies in whiteleg shrimp (*Penaeus vannamei*) [21], pacu (*Piaractus mesopotamicus*) [22], and Nile tilapia (*Oreochromis niloticus*) [23,24,25], where lower feed conversion ratios were consistently reported in BFT systems. The improvement is likely due to the utilization of bioflocs as supplemental feed, which can contribute up to 50% of the diet by providing microbial protein and organic nutrients [26,27]. These results indicate that BFT-based systems not only enhance apparent feed efficiency but also promote more sustainable feed resource use [28].

In addition to fish performance, the growth of caipira lettuce was higher in BFT-AP system compared with RAS-AP. This species is widely cultivated due to its rapid growth, high market acceptance, and adaptability to aquaponic systems [12,18,20,29,30,31]. The enhanced caipira lettuce growth in BFT-AP can be explained by higher concentrations of EC, TDS, essential macronutrients (K, Ca, P, S) and overall ionic content in the BFT-AP water. Moreover, the addition of molasses to maintain C/N ratio in BFT not only supplies an organic carbon source [28] but also contributes minerals such as K, Ca, Mg, and P [20], enriching the nutrient profile of system and potentially replacing artificial nutrient solutions. The elevated nutrient levels in aquaponics systems (BFT-AP and RAS-AP) also suggest active ion uptake and utilization by plants, promoting a more balanced ecological cycle between fish and crops. The inferior caipira lettuce production observed in RAS-AP compared to BFT-AP is likely attributable to the nutrient removal characteristics of RAS. The mechanical filtration (e.g., drum filter) effectively removes fine particulates and organic matter, which also contain plant-available nutrients such as phosphorous, trace minerals, and dissolved organic nitrogen.

Water quality management including parameters such as temperature, DO, EC, TDS, pH, and nitrogen compounds is critical for sustainable aquaculture operation. In this study, temperature was maintained at 25 °C, within the optimal range for eels (22.5–26.5 °C) [32,33], and DO levels (6.61–8.68 mg/L) were appropriate for feeding activity and metabolism [34]. EC and TDS were higher in BFT and BFT-AP systems, indicating greater nutrient accumulation derived from microbial and organic matter decomposition [34,35]. These elevated EC and TDS levels, particularly in BFT-AP, likely created a nutrient-rich environment beneficial for plant growth [20,36]. The pH range (6.61–7.80) was well maintained through sodium bicarbonate addition, which is optimal for nitrifying bacterial activity [37,38,39].

Nitrogenous compounds such as TAN and NO_2_^−^-N remained below 0.52 mg/L and 0.82 mg/L, respectively, both within safe levels for eel culture [40,41]. Although nitrate toxicity is relatively low, excessive accumulation can still impact fish health [14]; therefore, maintaining appropriate denitrification processes and water exchange is essential [40,41,42]. The NO_3_^−^-N concentrations observed in this study were within the recommended range [40], indicating effective system management.

Nevertheless, this study has some limitations. The experimental period was relatively short (four weeks), which may not fully represent long-term system stability or biofloc community succession. Although this study focused on short-term performance, it is important to note that BFT systems may experience nutrient and ion accumulation, increased oxygen demand, and solids management challenges during long-term operation. These considerations highlight the need for extended monitoring and future long-term evaluations to fully assess the system’s practicality in real farming settings. One plant species (*L. sativa*) and one fish species (*A. japonica*) were tested, limiting the generalization of results to other aquaponic species combinations. Additionally, microbial community structure and nutrient transformation dynamics were not analyzed in detail, which could provide deeper insight into the underlying mechanisms of improved performance. Future studies should, therefore, focus on long-term trials encompassing multiple fish and plant species, microbial community profiling, and nutrient cycling analysis.

Moreover, scaling up aquaponic BFT systems under commercial conditions and evaluating their economic feasibility and environmental impact would provide valuable information for practical application and policy development.

In addition, one important limitation of this study is that each treatment was implemented in a single hydraulic loop, resulting in shared water and filtration among tanks within the same treatment. Consequently, tanks represented subsamples rather than true replicates, and inferential statistics were not applied to avoid pseudoreplication. The findings should, therefore, be interpreted as system-level responses, and future studies should include multiple independent modules per treatment to allow mixed-model or factorial analysis.

## 5. Conclusions

This study demonstrated that integrating aquaponics into BFT and RASs enhance the growth performance and apparent feed efficiency of Japanese eel (*A*. *japonica*) and improved plant productivity, particularly in BFT-AP system. Higher EC, TDS, and nutrient ion concentrations in BFT-AP water likely contributed to superior caipira lettuce growth, while water quality parameters in all systems remained within the optimal range for eel culture.

These results indicate that combining aquaponics with biofloc technology creates a more resource-efficient and ecologically balanced aquaculture environment. Overall, the findings suggest that aquaponic BFT systems can serve as a promising sustainable aquaculture approach for eel production. Further research should evaluate long-term system performance, microbial community dynamics, and nutrient recycling efficiency to optimize these integrated systems for broader commercial use.

## Figures and Tables

**Table 1:** Growth performance of eels rearing with BFT, BFT-AP, RAS, RAS-AP for 4 weeks.

	Initial Weight (g/Fish)	Survival (%)	Final Weight (g/Fish)	Weight Gain (g/Fish)	SGR (%/Day)	CF	FE
BFT	110.5 ± 0.0	100.0 ± 0.0	159.2 ± 3.7	48.7 ± 3.8	1.30 ± 0.08	0.13 ± 0.0	0.67 ± 0.05
BFT-AP	110.5 ± 0.0	97.2 ± 2.1	170.9 ± 5.4	60.5 ± 5.4	1.56 ± 0.11	0.14 ± 0.0	0.76 ± 0.04
RAS	110.5 ± 0.0	97.8 ± 1.8	157.3 ± 4.2	46.8 ± 4.2	1.26 ± 0.10	0.13 ± 0.0	0.59 ± 0.04
RAS-AP	110.5 ± 0.0	91.1 ± 2.6	166.1 ± 4.9	55.6 ± 4.9	1.46 ± 0.10	0.13 ± 0.0	0.56 ± 0.02

Values are presented mean ± SD of four subsample tanks within each shared system (system = 1). No inferential statistical tests were performed due to the shared system structure. SGR, specific growth rate; CF, condition factor; FE, apparent feed efficiency.

**Table 2:** Major ion and nutrient concentrations of rearing water after 4-week experiment in BFT, BFT-AP, RAS, RAS-AP systems.

Contents (mg/L)	BFT	BFT-AP	RAS	RAS-AP
Cl	25.35 ± 0.78	28.37 ± 1.15	20.03 ± 0.47	19.93 ± 0.59
SO_4_	33.25 ± 8.13	30.37 ± 8.52	17.77 ± 3.93	21.27 ± 4.93
Total-N	25.41 ± 3.89	21.82 ± 0.58	5.28 ± 2.12	3.96 ± 1.49
Total-P	7.79 ± 1.43	5.61 ± 2.00	0.58 ± 0.18	0.43 ± 0.03
Ca	39.10 ± 5.86	38.50 ± 0.00	28.33 ± 0.15	26.97 ± 0.15
Cu	0.01 ± 0.01	0.04 ± 0.02	0.04 ± 0.01	0.02 ± 0.00
Fe	0.16 ± 0.03	0.08 ± 0.01	0.01 ± 0.01	0.02 ± 0.00
K	14.20 ± 0.99	7.27 ± 5.11	1.80 ± 0.00	0.87 ± 0.06
Mg	8.55 ± 1.06	7.10 ± 0.53	5.23 ± 0.06	4.90 ± 0.00
Mn	0.00 ± 0.00	0.00 ± 0.01	0.00 ± 0.00	0.00 ± 0.00
Na	66.15 ± 4.60	36.93 ± 5.61	18.70 ± 0.17	17.77 ± 0.31
Zn	0.09 ± 0.02	0.11 ± 0.15	0.00 ± 0.00	0.00 ± 0.00
Si	2.05 ± 0.13	2.06 ± 0.02	2.23 ± 0.01	2.19 ± 0.01

Values are presented mean ± SD of three subsample tanks within each shared system (system = 1). No inferential statistical tests were performed due to the shared system structure.

**Table 3:** Major ion and nutrient concentrations of caipira lettuce (*L. sativa*) after 4-week experiment in BFT-AP and RAS-AP systems.

Contents (mg/L)	BFT-AP	RAS-AP
Cl	25.85 ± 0.75	17.90 ± 1.56
SO_4_	22.50 ± 0.57	18.05 ± 7.14
Total-N	5.31 ± 1.99	4.56 ± 0.60
Total-P	3.46 ± 0.16	0.30 ± 0.12
Ca	33.35 ± 1.48	26.15 ± 0.07
Cu	0.03 ± 0.00	0.01 ± 0.00
Fe	0.07 ± 0.01	0.02 ± 0.00
K	2.85 ± 0.07	0.50 ± 0.00
Na	40.05 ± 1.48	17.35 ± 0.07
Si	2.03 ± 0.09	2.12 ± 0.05

Values are means ± SD (n = 2).

## Data Availability

Data are available upon reasonable request.
